# New Flow Cytometric Methods for Monitoring STAT5 Signaling Reveal Responses to SARS-CoV-2 Antigen-Specific Stimulation in FOXP3+ Regulatory T Cells also in Patients with Advanced Chronic Lymphocytic Leukemia

**DOI:** 10.3390/bios13050539

**Published:** 2023-05-11

**Authors:** Zlatko Roškar, Mojca Dreisinger, Primož Tič, Evgenija Homšak, Sebastjan Bevc, Aleš Goropevšek

**Affiliations:** 1 Department of Haematology, University Medical Centre Maribor, 2000 Maribor, Slovenia; zlatko.roskar@ukc-mb.si (Z.R.); mojca.dreisinger@ukc-mb.si (M.D.); 2 Department of Laboratory Diagnostics, University Medical Centre Maribor, 2000 Maribor, Slovenia; primoz.tic@ukc-mb.si (P.T.); evgenija.homsak@ukc-mb.si (E.H.); ales.goropevsek@ukc-mb.si (A.G.); 3 Faculty of Medicine, University of Maribor, 2000 Maribor, Slovenia; 4 Department of Nephrology, University Medical Center Maribor, 2000 Maribor, Slovenia

**Keywords:** STAT signaling, Treg, chronic lymphocytic leukemia, SARS-CoV-2

## Abstract

Increased frequency of CD4^+^CD25^+^ regulatory T-cells (Treg) has been associated with disease progression in chronic lymphocytic leukemia (CLL). Flow cytometric methods, which allow for the simultaneous analysis of their specific transcription factor Foxp3 and activated STAT proteins, together with proliferation can help to elucidate the signaling mechanisms driving Treg expansion and suppression of FOXP3- conventional CD4^+^T-cells (Tcon). Herein, we first report a novel approach in which STAT5 phosphorylation (pSTAT5) and proliferation (BrdU-FITC incorporation) could be analyzed specifically in FOXP3+ and FOXP3- responding cells after CD3/CD28 stimulation. The addition of magnetically purified CD4^+^CD25^+^ T-cells from healthy donors to cocultured autologous CD4^+^CD25^−^ T-cells resulted in suppression of Tcon cell cycle progression accompanied by a decrease in pSTAT5. Next, a method using imaging flow cytometry is presented for the detection of cytokine-dependent pSTAT5 nuclear translocation in FOXP3-expressing cells. Finally, we discuss our experimental data obtained by combining Treg pSTAT5 analysis and antigen-specific stimulation with SARS-CoV-2 antigens. Applying these methods on samples from patients revealed Treg responses to antigen-specific stimulation and significantly higher basal pSTAT5 in CLL patients treated with immunochemotherapy. Thus, we speculate that through the use of this pharmacodynamic tool, the efficacy of immunosuppressive drugs and their possible off-target effects can be assessed.

## 1. Introduction

Flow cytometry immunophenotyping is a powerful technology that can be used to identify cell membrane antigens and remains an indispensable tool for the diagnosis, classification, staging, and monitoring of hematologic neoplasms [1]. Chronic lymphocytic leukemia (CLL) is the most common form of leukemia in Western countries [2]. It is characterized by the accumulation of monoclonal B-lymphocytes in bone marrow, lymphoid organs, and peripheral blood, which is accompanied by expansion of the transcription factor forkhead box protein 3 (FOXP3) expressing regulatory T-cells (Treg) [3,4,5,6,7].

The negative regulation of an immune response, as mediated by Tregs, may not only hinder effective immune responses against leukemic B-cells, but also prevent pathogen clearance during infections [8,9,10]. In addition, CLL patients which are treated with B-cell-targeting therapies, such as the anti-CD20 antibody rituximab or the Bruton tyrosine kinase (BTK) inhibitor ibrutinib, have a decreased serological response to various vaccines [11]. The most recent studies have shown that although the humoral immune response to SARS-CoV-2 vaccine is severely impaired in patients treated with rituximab or ibrutinib, a significant fraction of these patients mount a SARS-CoV-2-specific T-cell response [12,13]. The cellular response to vaccination inversely correlated with the amount of B cells in ibrutinib-treated CLL groups, suggesting that the generation of the cellular immune response to the vaccine is hampered by the disease burden [13]. Of note, increased frequencies of Treg cells significantly correlate with disease burden and prognosis (advanced Rai or Binet stages, shorter time to first treatment) [14,15,16,17,18]. Therefore, in CLL, where the balance between immune activation and suppression is skewed, Tregs could be attractive pharmacological targets.

Tregs, which also highly express CD25 [i.e., interleukin (IL)-2α receptor], constitute an essential mechanism of peripheral T-cell tolerance. This was first demonstrated in Treg-depleted animal models, which develop diffuse autoimmunity [19]. Recent trials emphasized the importance of directly assessing the human immune response, and that not all of what we learn, for example, in rodents can be directly translated to humans [20,21]. Thus, there is a need for the development of tools and assays to directly assess the human immune system and to predict its responses to novel therapeutic entities. In addition, there have been increased calls for advanced in vitro or even ex-vivo multi-cellular infection and drug discovery models that could provide reliable and valuable tools correlative to in vivo [22].

Thornton and Shevach pioneered the in vitro suppression assay that mimics the function of Tregs in vivo. They have shown that, when cocultured with polyclonally stimulated CD4^+^CD25^−^ responder cells, the CD4^+^CD25^+^ cells markedly suppressed proliferation by specifically inhibiting the production of IL-2 [23]. Homeostatic common gamma-chain cytokines, such as IL-2 and IL-7, act on both Treg and FOXP3− conventional CD4^+^ T-cells (Tcon) through their intracellular signaling pathways that lead from surface receptors, mainly through STAT5 proteins (Signal Transducers and Activator of Transcription 5), which upon being activated–phosphorylated at specific tyrosine residues, translocate to the nucleus and control numerous gene programs also controlling their proliferation [24].

We have shown before that detection of intracellular signaling proteins with phosphor-specific flow cytometry is a valuable tool to monitor the JAK-STAT signaling pathway immediately ex vivo. Observations of our longitudinal studies implicate basal activation of STAT proteins in whole blood CD4^+^ T-cells as a possible marker of severe disease course in patients with systemic autoimmune disease (SLE) [25,26].

Herein, we first report a novel approach in which we could directly monitor STAT signaling and proliferation (DNA synthesis by BrdU incorporation), combined with FOXP3 expression, in CD4^+^ T-cells after CD3/CD28 stimulation. In this manner we could assess differences in STAT activation between the proliferating and non-proliferating CD4^+^ T-cells. In addition, for the first time, STAT signaling and proliferation could be analyzed simultaneously and specifically in FOXP3+ and FOXP3− responding T-cells in a unique in vitro assay. The addition of CD25^+^CD4^+^ to cocultured CD25^−^CD4^+^ cells from healthy donors was able to suppress proliferation (cell cycle progression) in responding FOXP3− Tcon cells. Consistent with a role of IL-2 signaling in FOXP3− Tcon cell cycle progression, we also found significantly lower Tcon levels of IL-2 dependent STAT5 phosphorylation (pSTAT5) in coculture with CD4^+^CD25^+^ cells.

Next, the phosphoflow method combined with imaging flow cytometry is presented for the detection of cytokine-dependent pSTAT5 and nuclear translocation in FOXP3 expressing cells after ex-vivo stimulation with recombinant human IL-2. Such novel approaches confirmed higher IL-2 induced responses, as well as pSTAT5 nuclear localization in FOXP3+ cells.

Finally, we show that the results of a novel flow cytometric-based assay, which measures Treg-specific STAT5 signaling responses to whole blood SARS-CoV-2 spike antigen stimulation, reflect those obtained using other traditional methods of antigen-specific T-cell analysis, such as the activation-induced cellular marker (AIM) assay.

Applying newly developed methods on whole blood samples from patients with CLL revealed CD25^+^FOXP3+ Treg responses to the stimulation, even in patients with advanced disease. In addition, significantly higher basal/unstimulated pSTAT5 levels were also found in FOXP3+ relative to the Tcon subpopulation of CD4^+^ T-cells from patients treated with chemoimmunotherapy (CIT).

## 2. Materials and Methods

### 2.1. Study Population

Thirty-nine untreated consecutive patients meeting the diagnostic criteria for CLL [27] were enrolled into the single-center, prospective cohort study.

Malignant disease burden in patients with CLL was assessed with Binet staging [27,28]. The inclusion criteria for the first part of our study was Binet stage C, while the exclusion criteria were Binet stage A and B. Whole blood samples (*n* = 36) from twenty patients at the start of therapy at enrollment (Binet stage C) were used for analysis of STAT signaling ex vivo before (*n* = 11) or during subsequent therapy with either BTK inhibitor (ibrutinib, *n* = 14) or CIT (combination of bendamustine or fludarabine and cyclophosphamide with rituximab, *n* = 11). Two of the patients enrolled in the first part of our study together with ten additional patients with CLL (regardless of the stage of the disease) were included in the second part of our study, where the Ag-specific response was analyzed. Relevant demographic and clinical data for the recruited patients are shown in Table 1. The patients were double vaccinated with BNT162b2 mRNA COVID-19 vaccine from 26 to 28 months ago. Some of them had also recovered from recent SARS-CoV-2 infection within a few months.

Blood samples for in vitro analyses were obtained from healthy adults (*n* = 12) with no history of allergy, acute infections, autoimmune disorders, or medications that affect the immune system.

Blood samples for the SARS-CoV-2 antigen-specific pSTAT5 and AIM assay were also obtained from 12 healthy laboratory personnel either at 26 to 32 months after the second dose of the BNT162b2 mRNA COVID-19 vaccine and/or following recovery from SARS-CoV-2 infection.

Participants were designated as naïve or previously infected for SARS-CoV-2 based on a positive PCR and/or serology at any time. For vaccination status, participants were designated as unvaccinated or vaccinated according to self-reported status.

### 2.2. Stimulation, Fixation, and Permeabilization for STAT5 Signaling Analysis

Preparation of samples for flow cytometry analysis of basal and cytokine-induced STAT5 phosphorylation ex vivo was conducted in EDTA anticoagulated whole blood. Typically, 100 µL of sample in a round bottom polystyrene test tube (Falcon^®^ 5 mL) was treated with 100 ng/mL IL-2 (Peprotech, Rocky Hill, NJ, USA) for 15 min at 37 °C in a water bath, or left untreated.

Phosphorylation in whole blood samples was stopped by fixation for 10 min using 2 mL of BD Phosflow Lyse/Fix Buffer (BD Biosciences, San Jose, CA, USA). Samples were then centrifuged at 300× *g* for 7 min and cells were permeabilized by incubation in 1 mL of ice-cold BD Perm Buffer III (BD Biosciences) for 30 min.

For STAT5 signaling analysis of peripheral blood mononuclear cells (PBMCs) and isolated CD4^+^ T-cells, the preparation of cells was the same as described above for whole blood, except for fixation of cells; BD Cytofix Fixation Buffer (BDPharmingen, BD Biosciences, San Jose, CA, USA) was used.

In selected experiments, samples were incubated with a neutralizing antibody, anti-IL-2 (2 µg/mL, clone MQ1-17H12, BD Biosciences), for 30 min at 37 °C before fixation.

### 2.3. Intracellular Staining and Flow Cytometry Analysis

After permeabilization, samples were centrifuged at 300× *g* for 5 min, followed by two consecutive washes with 2 mL of PBS. Cells were then stained for 30 min in 100 µL of stain buffer (PBS/2% FBS) with anti-CD45-PerCP (5 μL, clone 2D1), BV786 (1 μL, clone HI30) or APC-Cy7 (5 μL, clone 2D1), anti-CD3-FITC (10μL, clone UCHT1), BV650 (3 μL, clone SK7), or PerCP (20 μL, clone SK7), and antibodies recognizing specific phosphorylated STAT5 tyrosine: pSTAT5 (Y694)-Alexa647 (10 μL, clone 47) or PE (10 μL, clone 47) (all BD Biosciences). For multiparametric immunophenotyping experiments, cells were simultaneously stained at room temperature with antibodies specific for T-cell-subsets: anti-CD4-PECy7 (5 μL, clone SK3) or BV750 (2 μL, clone SK3), anti-CD25-PE (10 μL, clone 2A3), BV421 (3 μL, clone 2A3) or APC (5 μL, clone 2A3), and anti-FOXP3-Alexa 488 (10 μL, clone 259D/C7) or PE (10 μL, clone 259D/C7) (all BD Biosciences). After the final wash with 2 mL of stain buffer, cells were analyzed on LSR II or FACSymphony A3 Flow Cytometer (Beckton Dickinson, Franklin Lakes, NJ, USA) using FACSDiva software (Becton Dickinson) and FlowJo (TreeStar, Ashland, OR, USA, now part of BD Biosciences). The median fluorescence intensity (MFI) of the pSTAT5-specific signal was measured. The gate for CD25^+^ was set according to a ‘fluorescence-minus-one plus isotype’ (FMO+I) control in which the same antibodies were used for staining as in the full stain, except for the anti-CD25 antibody, which were substituted with an isotype control antibody labeled with the same fluorochrome.

### 2.4. In Vitro Assay with Combined STAT5 Signaling and Cell Cycle Analysis

PBMCs were separated from peripheral blood by density gradient centrifugation with Histopaque 1077 (Sigma-Aldrich). Purified CD4^+^ T-cell populations were prepared from PBMCs using negative selection by magnetic cell sorting (BDImag Human CD4 T Lymphocytes Enrichment Set–DM, BDBiosciences). CD4 T-cells were further separated into CD25^+^ and CD25^−^ populations (BD IMag™ Anti-Human CD25 Magnetic Particles), anti-CD3/CD28 stimulated with plate-bound anti-CD3 (5 μg/mL), and soluble anti-CD28 (1 μg/mL) in 96-well plates and cultured in complete RPMI (RPMI 1640 plus L-glutamine, penicillin, streptomycin; Invitrogen) with 10% fetal calf serum (Invitrogen) in a 1:1 ratio. In total, 2 × 10^5^ cells were seeded per well, consisting of enriched CD4^+^CD25^+^ cells, autologous CD4^+^CD25^−^ T-cells as responder Tcon cells alone or a mixture of both in a 1:1 ratio, and anti-CD3/CD28 stimulated for 2 days in CO_2_ incubator. Cells were pulsed with 5-bromo-2′-deoxyuridine (BrdU, 10 μM, BrdU Flow Kit, BDPharmingen) for one hour before harvest, prepared as for the STAT5 signaling analysis described above with some changes in protocol due to additional treatment of cells with DNase (300 μg/mL, BrdU Flow Kit, BDPharmingen). For fixation of cells, an equal volume of Cytofix Fixation Buffer (BDPharmingen) was added immediately to wells of the cell culture plate. After mixing and further addition of half medium and half fixative to 1 mL, cells were transferred to round bottom polystyrene test tube (Falcon^®^ 5 mL) and incubated for 10 min at 37 °C in a water bath. After centrifugation at 300× *g* for 5 min and permeabilization in 1 mL of 10× diluted ice-cold BD Perm Buffer III (BD Biosciences) for 30 min, samples were again centrifuged at 300× *g* for 5 min, followed by two consecutive washes with 2 mL of sterile-filtered PBS. Samples were than incubated in 500 µL sterile PBS with 2% FBS for at least 15 min. After centrifugation at 300× *g* for 5 min, 100µL of DNase (30 µg) was added and samples were incubated for 1 h at 37 °C, followed by another wash with 1 mL of PBS. Anti-BrdU was diluted 1:50 with Stain Buffer (PBS with 2% FBS). Cells were fluorescently labelled with anti-CD4-PECy7, anti-FOXP3-PE, anti-BrdU-FITC, and anti-pSTAT5-Alexa647 (all BD Biosciences). Antibodies were added and resuspended in 50 µL of diluted anti-BrdU solution and samples were incubated for 30 min in the dark at room temperature. After the final wash with 2 mL Stain Buffer and centrifugation at 300× *g* for 5 min, cells were stained with 7-AAD nuclear stain (0.25 μg, BD Pharmingen) in 500 µL of Stain Buffer at room temperature for 10 min. Samples were acquired on a BD LSRII flow cytometer and data analyzed using FlowJo software (Tree Star) and FacsDiva software (Becton Dickinson, San Jose, CA, USA). The purity of magnetically-selected CD4^+^ T-cells and their CD25^+^/CD25^−^ subsets was analyzed in the preliminary experiments by staining with anti-CD45 PerCP, CD3 FITC, CD25 PE, and CD4 PE-Cy7 antibodies (antibody clones described above, all from BD Biosciences), which were analyzed on the same instrument.

### 2.5. Preparation of Cells for Intracellular Cytokine, FOXP3, and STAT5 Signaling Analysis

After PBMCs were separated from peripheral blood by density gradient centrifugation with Histopaque 1077, purified CD4^+^ T-cell populations were prepared by magnetic cell sorting as described above. They were anti-CD3/CD28 stimulated with plate-bound anti-CD3 and soluble anti-CD28 in 96-well plates and cultured as described above for 24 h. Intracellular cytokine staining of interleukin-2 (IL-2) or interferon gamma (IFN-g) was performed after the stimulation while incorporating GolgiStop (BD Biosciences) for the last 6 h. After fixation and permeabilization, as described above, samples were stained at room temperature with anti-CD4-PECy7, anti-FOXP3-FITC, anti-pSTAT5-Alexa647, and anti–IL-2 PE (20 μL clone MQ1-17H12) or anti–IFNg PE (20 μL, clone B27) (all BD Biosciences). Cells were acquired on a BD LSRII flow cytometer and data analyzed using FlowJo software (Tree Star) and FacsDiva software (Becton Dickinson, San Jose, CA, USA). The gate for IL−2^+^ and IFN-g+ cells was set according to a ‘fluorescence-minus-one plus isotype’ (FMO+I) control in which the same antibodies were used for staining as in the full stain, except for the anti-IL-2 or anti-IFNg antibody, which was substituted with an isotype control antibody labeled with the same fluorochrome. The ancestry gating strategy for analysis of separated CD4^+^ T-cells is shown on Supplementary Figure S1. Samples were acquired on a BD LSRII flow cytometer and data was analyzed using FlowJo software (Tree Star) and FacsDiva software (Becton Dickinson, San Jose, CA, USA).

### 2.6. Analysis of Cytoplasmic and Nuclear Localization of Transcription Factors FOXP3 and pSTAT5

Whole blood samples were prepared and stained for CD3, FOXP3, CD4, and phosphotyrosine-STAT5, as described above, and counterstained with 20 ng/mL 7-AAD (BD Biosciences). Image files of cells were collected for each sample using the ImageStreamX imaging flow cytometer (Amnis, Seattle, WA, USA) and were analyzed using IDEAS software (Amnis), as described before [29]. In-focus single cells were identified by gating on 7-AAD-positive events with high nuclear aspect ratios (minor to major axis ratio, a measure of circularity) and high nuclear contrast (as measured by the Gradient Max feature). Among lymphocytes (low side scatter/low area cells), FOXP3 expressing cells were gated. Granulocytes with high side scatter were also gated on side scatter versus area dot plot. Nuclear localization of phosphotyrosine-STAT5 within these cells was measured using the similarity score, which quantifies the correlation of pixel values of the nuclear and phosphotyrosine-STAT5 images on a per-cell basis [30]. If the transcription factor is nuclear, the two images will be similar and have large positive values. If it is cytoplasmic, the two images will be anti-similar and have large negative values. Events with positive values >1 had visually apparent nuclear distributions of transcription factors and were gated to quantify the percentage of cells with nuclear-localized phosphotyrosine-STAT5 within the FOXP3+ lymphocyte population.

### 2.7. Whole Blood Flow Cytometric SARS-CoV2-Specific pSTAT5 Assay

We evaluated STAT5 signaling responses to whole blood antigen-specific stimulation by using heparinized antigen tubes from the QuantiFERON SARS-CoV-2 kit (Qiagen, Hilden, Germany). This is an IFNg release assay, which contains heparinized antigen tubes that allow both the collection of whole blood and stimulation of lymphocytes with a combination of two antigen peptides specific to SARS-CoV-2 (SARS-CoV-2 Ag1 and Ag2). The SARS-CoV-2 Ag1 tube contains CD4^+^ epitopes derived from the S1 subunit of the spike protein and the SARS-CoV-2 Ag2 tube contains CD4^+^ and CD8^+^ epitopes from the S1 and S2 subunits of the spike protein.

Whole blood samples were collected directly into the assay collection tubes, shaken, and incubated for 16–24 h.

Whole-blood aliquots (120 μL) were withdrawn from Nil (negative control) and from the two Ag tubes (60 μL each, mixed together) of the QuantiFERON SARS-CoV-2 kit before centrifugation and prepared for STAT5 signaling analysis. Phosphorylation in samples was stopped by fixation for 10 min using 2 mL of BD Phosflow Lyse/Fix Buffer (BD Biosciences, San Jose, CA, USA). Samples were then centrifuged at 300× *g* for 7 min and cells were permeabilized with incubation in 1 mL of ice-cold BD Perm Buffer III (BD Biosciences) for 30 min, as described above, for the whole blood samples. After permeabilization, samples were centrifuged at 300× *g* for 5 min, followed by two consecutive washes with 2 mL of PBS. Cells were then stained at room temperature for 30 min in 100 µL of stain buffer (PBS/2% FBS) with the following combination of anti-human fluorescent monoclonal antibodies: CD3 BV786 (2 μL, clone UCHT1), CD4 BV750, CD45RA PE-Cy7 (3 μL, HI100), CD25 BV421, FOXP3 FITC, and anti-pSTAT5-Alexa647 (all Becton Dickinson, San Jose, CA, USA). Cells were acquired on the FACSymhony A3 flow cytometer and data was analyzed using FlowJo software (Tree Star) and FacsDiva software (Becton Dickinson, San Jose, CA, USA).

### 2.8. Activation-Induced Marker (AIM) Assay

The AIM assay was also performed with whole blood antigen-specific stimulation by using heparinized antigen tubes from the QuantiFERON SARS-CoV-2 kit (Qiagen).

Whole blood samples were collected directly into the assay collection tubes, shaken, and incubated for 16–24 h, as described above. Whole-blood aliquots (120 μL) were withdrawn from Nil (negative control) and from the two Ag tubes (60 μL each, mixed together) of the QuantiFERON SARS-CoV-2 kit before centrifugation, and stained for 15 min at room temperature with the following combination of anti-human fluorescent monoclonal antibodies: CD3 BV786, CD4 BV750, CD134 BB700 (3μL, Clone L106), and CD25 BV421 (all Becton Dickinson, San Jose, CA, USA). Incubation in 2 mL of FacsLyse solution (Becton Dickinson, San Jose, CA, USA) for 10 min was used to remove red blood cells. After centrifugation at 300× *g* for 5 min and washing with 2 mL of stain buffer, 0.5 mL samples were acquired on the FACSymhony A3 flow cytometer and data was analyzed using FlowJo software (Tree Star) and FacsDiva software (Becton Dickinson, San Jose, CA, USA). Cells were gated on the forward scatter/side scatter cell gate, FSC-A vs. FSC-H, to exclude doublets and then on the CD3+CD4^+^ gate for the quantification of CD25^+^OX-40+ SARS-CoV-2-specific CD4 T-cells.

### 2.9. Construct of the Presented Study and Analysis

**Figure 1:**
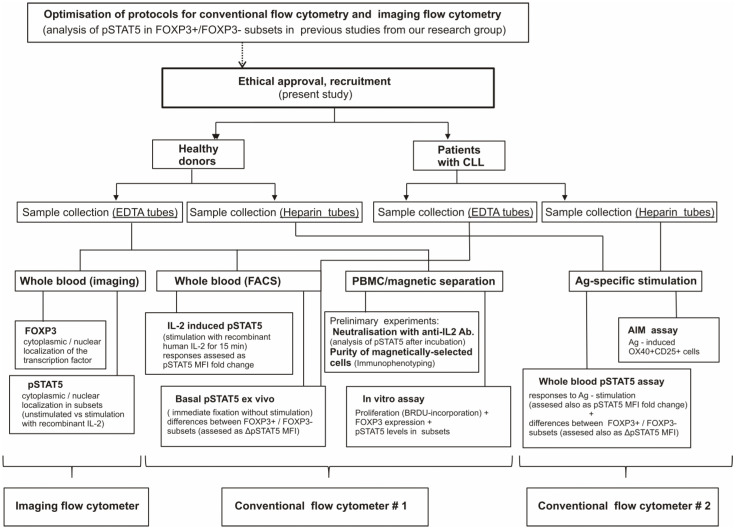
The study-flow diagram.

The study-flow diagram shows the construct of the study and all-associated laboratory analysis that were performed (Figure 1).

### 2.10. Statistical Analysis

Statistical analysis was performed using GraphPad Prism version 9 for Windows (GraphPad Software, San Diego, CA, USA). The Mann–Whitney test was used for the between-group comparisons, while for within-group comparisons, the Wilcoxon matched-pairs signed rank test was used. The Spearman correlation coefficient was calculated to investigate possible associations between variables, and *p* values less than 0.05 were considered significant.

## 3. Results

### 3.1. Flow Cytometry-Based In Vitro Assay Detects Increased IL-2-Dependent STAT5 Signaling in FOXP3+ Cells and Suppression of Cell Cycle Progression of Responding Tcon Cells

**Figure 2:**
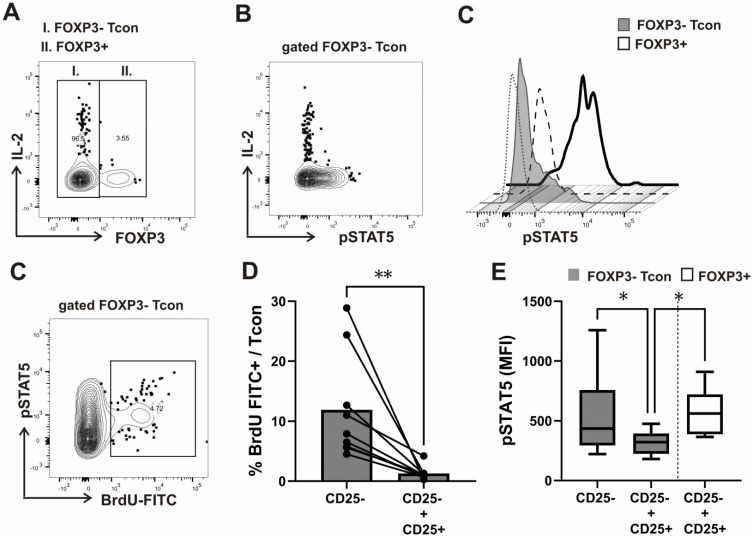
Intracellular IL-2 expression, proliferation, and STAT5 signaling analysis in FOXP3+ and FOXP3− conventional (Tcon) subsets of CD4^+^ T-cells after CD3/CD28 stimulation in vitro. (**A**) FOXP3 expression and STAT5 phosphorylation (pSTAT5) analysis, combined with intracellular cytokine staining of interleukin-2 (IL-2) or IFN-gamma was performed in purified CD4^+^ T-cells from healthy donors (*n* = 6) after anti-CD3/CD28 stimulation for 24 h with the addition of a protein transport inhibitor containing monesin for the last 6 h, as described in the materials and methods. Representative contour plot shows the levels of IL-2 expression in gated FOXP3+ versus FOXP3− conventional (Tcon) CD4^+^ T-cells (**B**). Representative contour plot depicts levels of IL-2 expression versus pSTAT5 in Tcon cells, gated as shown on Figure 2A. (**C**) During the in vitro assay, described in the materials and methods, STAT5 signaling and proliferation (cell cycle progression to S phase as determined by BrdU incorporation) was analyzed simultaneously and specifically in Tcon and FOXP3+ cells. Representative histograms of pSTAT5 levels after anti-CD3/CD28 stimulation for 48 h in coculture of CD4^+^CD25^−^ T cells with autologous CD4^+^CD25^+^ T cells isolated from healthy donor PBMCs are shown. Staining with FMO controls in FOXP3+ subset is shown with dashed and in FOXP3− Tcon subset with dotted lines. (**D**) Cell cycle analysis—gating and determination of % cells in S phase of cell cycle on bivariate distributions of BrdU content (FITC) versus pSTAT5 of gated FOXP3− Tcon cells. (**E**) Cumulative cell cycle progression (% of cells in S phase) analyzed in Tcon cells from healthy donors (*n* = 9), gated as outlined on Fig 1E when CD4^+^CD25^−^ cells were cultured alone as compared to coculture with CD4^+^CD25^+^ cells, added at a 1:1 ratio. Data are expressed as mean with SD. (**F**) Cumulative pSTAT5 levels (Median fluorescence intensity—MFI) analyzed in gated Tcon and FOXP3+ cells when CD4^+^CD25^−^ cells were cultured alone as compared to coculture with CD4^+^CD25^+^ cells isolated from PBMCs in healthy donors (*n* = 6). Tukey box (median, 25th to 75th percentiles) and whiskers plot. * *p* < 0.05, ** *p* < 0.01 with the Mann–Whitney test for the comparison between groups and the Wilcoxon rank-sum for within group comparisons.

First, we combined flow cytometric analysis of pSTAT5 with the analysis of intracellular IL-2 produced in response to anti-CD3/CD28 stimulation of CD4^+^ T-cells prepared by magnetic bead purification. In addition, by simultaneously assessing FOXP3 expression, we could show that intracellular IL-2 production was largely restricted to FOXP3- Tcon cells (Figure 2A and Figure S2). Moreover, pSTAT5 and IL-2 (Figure 2B and Figure S3), but not IFN-gamma (Supplementary Figure S4), expression in Tcon cells were also almost completely mutually exclusive.

It was shown before that mouse Tregs induce a marked decrease in IL-2-dependent STAT5 phosphorylation in Tcons by comparing two types of cell cultures in a classical in vitro suppression assay; effector Tcon cells alone undergoing activation by anti-CD3/anti-CD28 cross-linking and effector Tcon cells undergoing the same activation in the presence of Treg cells, purified as CD4^+^CD25^+^ cells [31]. The suppressor effect of CD4^+^CD25^+^ Treg in co-cultures with Tcon effector cells obtained by magnetic columns from healthy donors and activated by CD3/CD28 was successfully measured by a proliferation assay using BrdU incorporation before, however, in an ELISA test [32]. In addition, efficiency of CD4⁺CD25⁺ cell purification using magnetic columns from human samples had to be checked by flow cytometric confirmation of FOXP3 expression in suppressor T-cells [32]. We therefore used a novel flow cytometric approach in which STAT signaling and proliferation could be analyzed simultaneously and specifically in FOXP3+ cells and FOXP3− responding Tcons. An in vitro assay was performed as a combined pSTAT5 and cell cycle analysis in CD4^+^T-cells prepared by magnetic bead purification, separated into CD25^+^ and CD25^−^ populations, and stimulated with anti-CD3/anti-CD28 for 2 days. In this way, we were able to compare levels of STAT5 phosphorylation between Tcon and FOXP3 expressing cells (Figure 2C). In addition, as shown on the representative contour plot of gated FOXP3− Tcon cells (Figure 2D), such flow cytometric approach indeed also allowed the simultaneous analysis of pSTAT5 and Tcon cell proliferation (DNA synthesis by BRDU incorporation) during the in vitro assay. When CD4^+^CD25^−^ cells were cultured alone, FOXP3− Tcons from healthy donors entered the cell cycle; cells accumulated in S phase, as measured by BrdU-FITC incorporation following CD3/CD28 stimulation. When CD4^+^CD25^+^ cells from healthy donors were added at a 1:1 ratio, they suppressed such cell cycle progression, significantly lowering the percentage of Tcons entering the S phase in coculture with CD4^+^CD25^+^ cells than when they were cultured alone (Figure 2E). Consistent with the role of STAT5 signaling in Tcon cell cycle progression, pSTAT5 levels (assessed as the MFI—median fluorescence intensity) in Tcon cells were also significantly lower in coculture with CD4^+^CD25^+^ cells. However, pSTAT5 levels were significantly higher in FOXP3+ cells than in FOXP3− Tcon cells in coculture (Figure 2F).

**Figure 3:**
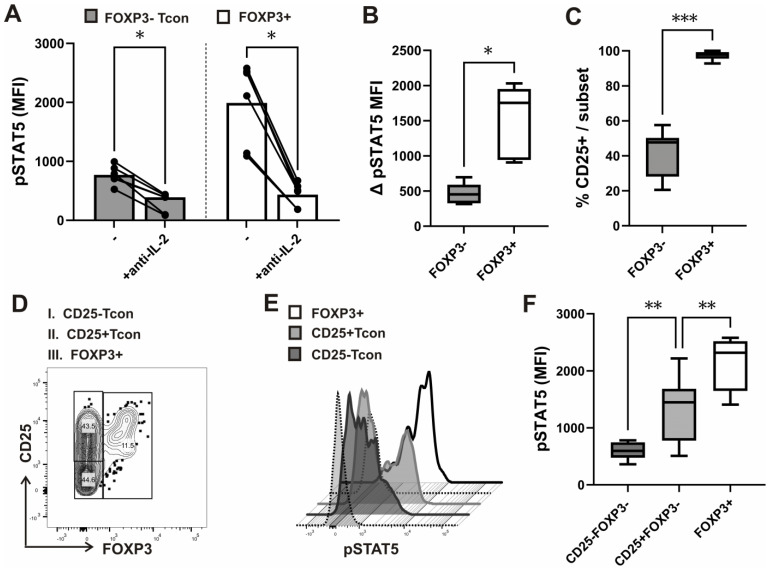
IL-2-dependent STAT5 signaling in CD25^+^ and CD25^−^ Tcon and FOXP3+ cells after anti-CD3/CD28 stimulation in vitro. (**A**) FOXP3 and CD25 expression combined with STAT5 phosphorylation (pSTAT5) analysis was performed in purified CD4^+^ T-cells from healthy donors (*n* = 6) after anti-CD3/CD28 stimulation for 24 h. Cumulative pSTAT5 levels (MFI) in gated FOXP3+ and FOXP3− Tcon subsets of CD4^+^ T-cells isolated from the same healthy donors, which were either incubated with neutralizing anti-IL2 antibodies or left without such treatment for the last 30 min of stimulation, as described in the materials and methods. Data are expressed as mean with SD. (**B**) Tukey box and whiskers plot of the decrease in pSTAT5 (expressed as delta pSTAT5 MFI) after treatment with anti-IL2 antibodies, as described for Figure 3A. (**C**) Tukey box and whiskers plot comparing percentages of CD25^+^ cells in the gated FOXP3+ subset and FOXP3− Tcon cells from healthy donors (*n* = 12). (**D**) Gating strategy of CD4^+^T lymphocytes stratified by FOXP3 and CD25 after CD3/CD28 stimulation, as described in the materials and methods. (**E**) Representative histograms of pSTAT5 levels after CD3/CD28 stimulation in CD25^+^FOXP3− and CD25^−^ FOXP3− Tcon subsets, compared to FOXP3+ cells. Staining with FMO controls in the FOXP3+ and FOXP3− subset is shown with dotted lines. (**F**) Cumulative pSTAT5 (MFI) in CD25^+^FOXP3− and CD25^−^ FOXP3− Tcon subsets, compared to FOXP3+ cells from healthy donors (*n* = 8). Tukey box and whiskers plot. * *p* < 0.05, ** *p* < 0.01, *** *p* < 0.001 with the Mann–Whitney test for the comparison between groups and the Wilcoxon rank-sum for within group comparisons.

We found that after anti-CD3/CD28 stimulation in vitro, pSTAT5 levels in Tcon and FOXP3+ cells were IL-2-dependent, as they were significantly decreased with neutralizing anti-IL-2 antibodies in both subsets (Figure 3A). In addition, the decrease in pSTAT5 (expressed as delta pSTAT5 MFI) after treatment with anti-IL2 antibodies was more significant in FOXP3+ cells than in Tcon cells (Figure 3B).

Thus, findings of our novel in vitro assay suggested that while suppression of cell cycle progression in responding FOXP3− Tcon cells is accompanied by a decrease in IL-2-dependent pSTAT5, FOXP3+ cells, possibly due to their higher sensitivity to IL-2, display higher levels of such STAT5 activation in coculture. Of note, in all CD3/CD28 stimulated samples, CD25 expression was found on more than 95% FOXP3+ cells compared to, on average, fewer than 50% Tcon cells (Figure 3C). In addition, FOXP3+ cells have shown significantly higher pSTAT5 even when compared to CD25^+^ Tcon cells, which displayed higher pSTAT5 levels than CD25^−^ Tcon cells (Figure 3D,E,F). Thus, while pSTAT5 levels in Tcon cells may be dependent on their levels of induced CD25, pSTAT5 levels in FOXP3+ cells could serve as a better read out of exposure to IL-2, produced at a given point of time during Tcon activation.

### 3.2. Nuclear Translocation and Subset-Specific Differences in STAT5 Phosphorylation Responses in an Ex Vivo Assay of IL-2-Induced Signaling

As IL-2-dependent differences in STAT5 signaling were found between subsets of CD4^+^ T-cells in vitro, we next performed flow cytometry analysis of cytokine-induced pSTAT5 ex vivo.

**Figure 4:**
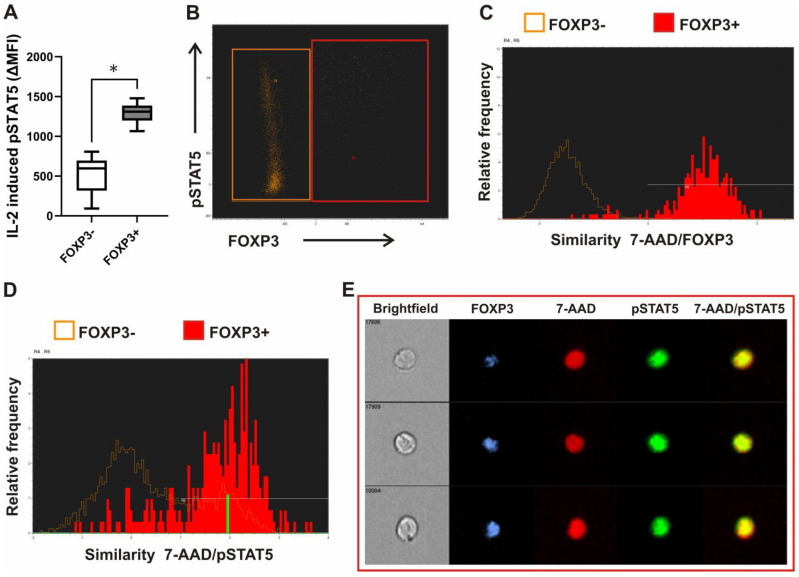
Recombinant human IL-2-induced STAT5 phosphorylation and nuclear translocation ex vivo in whole blood FOXP3+ CD4^+^ T-cells. (**A**) Leukocytes in blood from healthy donors (*n* = 6) were stimulated with recombinant human IL-2 for 15 min or left untreated. Tukey box and whiskers plot of the increase in pSTAT5 above untreated/basal levels after treatment with IL-2 (expressed as delta MFI) in gated FOXP3+ and FOXP3− CD4^+^ T cells. * is *p* < 0.05. (**B**) Leukocytes in whole blood from healthy donors were stimulated with IL-2 for 15 min and quantified for colocalization of FOXP3 and pSTAT5 within the 7-AAD stained nucleus using imaging flow cytometry. Lymphocytes were first gated as low SSC/low area cells. FOXP3+ and FOXP3− subset was gated as shown on the representative FOXP3 vs. pSTAT5 dot plot. (**C**,**D**) Representative histograms of 7-AAD/FOXP3 and 7-AAD/pSTAT5 similarity scores, respectively, in FOXP3+ (red) as compared FOXP3− cells (orange). (**E**) The example images of cells: pSTAT5 (green) is specifically localized to the nucleus stained with 7-AAD (red) in FOXP3 (blue) expressing cells. Brightfield images of cells are also shown.

When whole blood samples from healthy donors were treated with recombinant human IL-2 for 15 min, the pSTAT5 response, measured as the difference between basal (unstimulated) and stimulated levels (∆ pSTAT5 MFI), was significantly higher in the FOXP3+ subset of CD4^+^ T-cells than in FOXP3− Tcon cells (Figure 4A). In contrast, levels of fluorescence assessed with FMO (Fluorescence Minus One) controls were not significantly different between the two subsets (Supplementary Figure S5A). The level of cellular autofluorescence, spectral overlap, population spreading, and nonspecific binding may be determined with, but is not limited to, internal control populations or the use of FMO controls [33]. Since background levels in FMO controls are determined by undesirable antibody binding as well as spectral overlap, they cannot be used to specifically expose any type of undesirable binding [34]. Therefore, in our previous studies, in which we analyzed STAT5 phosphorylation in subsets of CD4^+^ T-cells with the same anti-pSTAT5 antibody, we also used an isotype control Ab [25,26]. Although some continue to use an isotype control Ab to determine/exclude non-specific binding, the isotype control is, according to the latest guidelines, not useful in this context [35]. Biological comparison controls are preferable for the quantification of phosphoproteins. Cells treated with neutralizing anti-IL-2 (Figure 3A,B) or anti-IL7 antibodies [25,26] can serve as a control in the analysis of common γ-chain cytokine-dependent STAT5 signaling. In addition, upon cytokine stimulation, phosphorylation was also quantified in this study by comparing fluorescence intensities between unstimulated controls and stimulated samples (Figure 4A).

Cytokine-dependent activation/phosphorylation of STAT5 results in the translocation of STAT5 homodimers to the nucleus. Infection with some viruses, however, was shown to perturb homeostatic cytokine signaling by impairing the access of pSTAT5 to the nuclear compartment [36]. Therefore, we used imaging flow cytometry to examine pSTAT5 localization in whole blood leukocytes after stimulation. The initial gating of lymphocytes was on low side scatter (SSC)/low area of cells. FOXP3 expressing and FOXP3 negative cells were gated as shown on Figure 4B. Nuclear localization of pSTAT5 and FOXP3 transcription factor within those cells was measured using the similarity score, which quantifies the correlation of pixel values of the nuclear stain 7-AAD and pSTAT5 images on a per-cell basis. If the transcription factor is nuclear, the two images will be similar and have large positive values. If it is cytoplasmic, the two images will not be similar and will have large negative values. Representative histograms of 7-AAD/FOXP3 and of 7-AAD/pSTAT5 similarity scores, correlating the 7-AAD nuclear stain with the FOXP3 and pSTAT5 signal, respectively, in the gated populations of lymphocytes are shown on Figure 4C,D. The higher the similarity score, the more nuclear localization was visualized in the sample images. Events with positive values >1 had visually apparent nuclear distributions of the transcription factor FOXP3. In addition, as also shown on representative images of such gated FOXP3+ events, pSTAT5 was localized to the nucleus after stimulation with recombinant human IL-2 in FOXP3 expressing lymphocytes (Figure 4E).

### 3.3. The Whole Blood Flow Cytometric SARS-CoV-2-Specific pSTAT5 Assay Shows Signaling Responses in the FOXP3+ Treg Subset Even in CLL Patients with Advanced Disease and on Chemoimmunotherapy

Bitar et al. demonstrated that the flow cytometry-based pSTAT5 assay represents an appropriate tool to quickly identify CMV-specific T cell proliferation [37]. As their assay measured pSTAT5 in gated CD3+ T-cells and still required mononuclear cell separation, our aim was to establish an assay that could measure subset specific STAT5 signaling responses to whole blood antigen specific stimulation. Results of the novel flow cytometric assay could reflect those obtained using other traditional methods of antigen-specific T cell analysis, such as the activation-induced cellular marker (AIM) assay, which detects cells that are activated as a result of antigen-specific stimulation by upregulation of activation-induced surface markers, such as the dual expression of OX40 (CD134) and CD25.

We evaluated the T-cell response by using heparinized antigen tubes from the QuantiFERON SARS-CoV-2 kit that allow both the collection of whole blood and stimulation of lymphocytes with a combination of two antigen peptides specific to SARS-CoV-2 (SARS-CoV-2 Ag1 and Ag2). The SARS-CoV-2 Ag1 tube contains CD4^+^ epitopes derived from the S1 subunit of the spike protein and the SARS CoV-2 Ag2 tube contains CD4^+^ and CD8^+^ epitopes from the S1 and S2 subunits of the spike protein.

Whole blood samples from healthy donors who were vaccinated with BNT162b2 mRNA COVID-19 vaccine and/or recovered from SARS-CoV-2 infection were used. In addition, we verified this assay also by analyzing pSTAT5 in both subsets from 12 patients with CLL who were double vaccinated with the BNT162b2 mRNA COVID-19 vaccine. Some patients had also recovered from recent SARS-CoV-2 infection and were positive when tested for anti-S antibodies.

**Figure 5:**
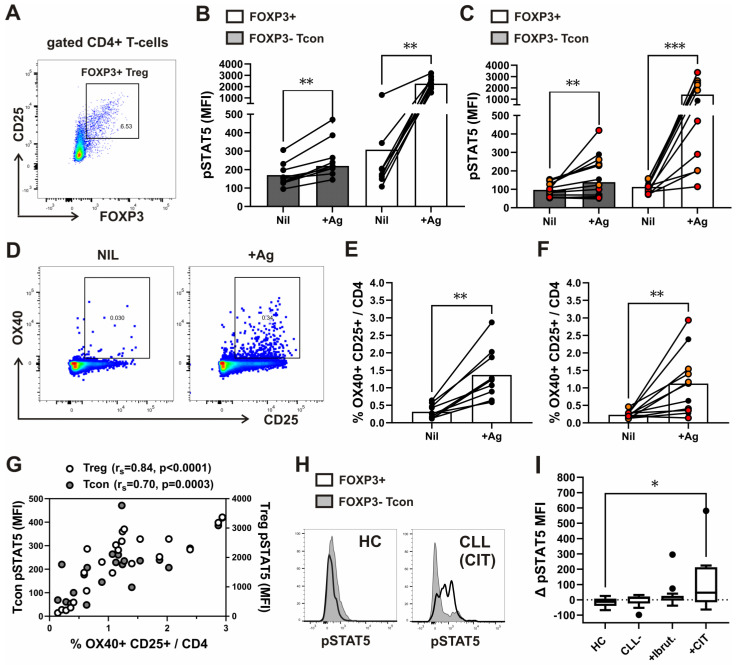
Basal/unstimulated pSTAT5 levels ex vivo and after whole blood stimulation with antigen peptides specific to SARS-CoV-2 in CD4^+^ T-cell subsets from healthy donors and patients with CLL. (**A**) Gating strategy for analysis of the FOXP3+CD25^+^ Treg subset among CD4^+^ T cells in whole blood samples, which were collected and incubated/stimulated in tubes from Quantiferon SARS-CoV-2 kit for 16–24 h, as described in the materials and methods. Representative FOXP3 vs. CD25 dot plot of gated CD4^+^ T-cells from a healthy donor is shown. (**B**) Cumulative Treg and Tcon pSTAT5 levels (MFI) in whole-blood aliquots withdrawn from negative control (Nil) and from the Ag tubes (+Ag). Symbols represent samples from healthy donors (*n* = 9). Data are expressed as mean with SD. (**C**) Cumulative Treg and Tcon pSTAT5 levels (MFI) in whole-blood aliquots withdrawn from negative control (Nil) and from the Ag tubes (+Ag). Symbols represent samples from patients with CLL (*n* = 12). Patients in Binet A stage of the disease are shown in black. While patients with advanced CLL in Binet C stage, treated with chemoimmunotherapy, are shown with red symbols, and patients before therapy or treated with kinase inhibitors with orange symbols. Data are expressed as mean with SD. (**D**) Gating strategy for the activation-induced cellular marker (AIM) assay, detecting the dual expressing OX-40+ CD25^+^ subset among CD4^+^ T cells in whole blood samples, which were collected and incubated/stimulated in tubes from Quantiferon SARS-CoV-2 kit for 16–24 h, as described in the materials and methods. Representative OX-40 vs. CD25 dot plots of gated CD4^+^ T-cells from the same healthy donor whole-blood aliquots withdrawn from negative control (Nil) and from the two Ag tubes mixed together (+Ag) are shown. (**E**) Cumulative percentages of the dual expressing OX-40+ CD25^+^ subset among CD4^+^ T cells in whole-blood aliquots withdrawn from negative control (Nil) and from the Ag tubes (+Ag). Symbols represent samples from healthy donors (*n* = 10). (**F**) Cumulative percentages of the dual expressing OX-40+ CD25^+^ subset among CD4^+^ T cells in whole-blood aliquots withdrawn from negative control (Nil) and from the Ag tubes (+Ag). Symbols represent samples from patients with CLL (*n* = 11). Patients in Binet A stage of the disease are shown in black. While patients with advanced CLL in Binet C stage, treated with chemoimmunotherapy, are shown with red symbols, and patients before therapy or treated with kinase inhibitors with orange symbols. Data are expressed as mean with SD. (**G**) Correlation between Treg and Tcon pSTAT5 levels (MFI) and percentages of the dual expressing OX-40+ CD25^+^ subset among CD4^+^ T cells in whole-blood aliquots withdrawn from the Ag tubes. (**H**) Representative histograms of basal/unstimulated pSTAT5 levels in the FOXP3+ and FOXP3− subset of CD4^+^ T-cells from patient with CLL in advanced stage Binet C, treated with chemoimmunotherapy, and from a healthy control, analyzed ex vivo, as described in the materials and methods. (**I**) Tukey box and whiskers plot compares differences in basal pSTAT5 levels (Δ pSTAT5 MFI) between the gated FOXP3+ and FOXP3− Tcon subset of CD4^+^ T-cells in whole blood samples from healthy controls (HC, *n* = 12), patients with advanced CLL (stage Binet C) before therapy (CLL, *n* = 11), and those treated with a BTK inhibitor (ibrutinib, *n* = 14) or chemoimmunotherapy (CIT, *n* = 11) * *p* < 0.05, ** *p* < 0.01, *** *p* < 0.001 with the Mann–Whitney test for the comparison between groups and the Wilcoxon rank-sum for within group comparisons. rs, Spearman rank correlation coefficient.

We analyzed the expression of AIM and compared it to pSTAT5 levels in FOXP3− Tcon and CD25^+^FOXP3+ Treg subsets (Figure 5A) from the same whole-blood aliquots, which were withdrawn from Nil (negative control) and from the two Ag tubes (mixed together) of the QuantiFERON SARS-CoV-2 kit.

STAT5 signaling responses, measured as the pSTAT5 MFI fold change (pSTAT5 MFI in stimulated tube divided by pSTAT5 MFI in control tube) [38], were significantly higher in Tregs than in Tcons, gated as shown on Figure 5A, from both healthy controls as well as patients with CLL (Supplementary Figure S5B). Levels of fluorescence assessed with FMO (Fluorescence Minus One) controls were not significantly different between the two subsets also in Ag-stimulated tubes (Supplementary Figure S6A). For cell signaling analysis, besides the FMO control, one must not neglect the biological negative controls, which were unstimulated cells also in our Ag-specific pSTAT5 assay (Supplementary Figures S5B and S6B).

In all samples from healthy donors, but not from all patients with CLL, higher pSTAT5 levels in the Tcon subset (Figure 5B,C), as well as higher percentages of CD4^+^ T-cells with dual expression of OX40 and CD25 (Figure 5D), were found following stimulation with the SARS-CoV-2 specific spike peptide mix (+Ag) compared to negative control (Nil) (Figure 5E,F). In addition, a significant correlation between pSTAT5 levels in both subsets and the percentage of OX40+CD25^+^ among CD4^+^ T-cells was found in antigen-stimulated, but not in control samples (Figure 5G). However, the correlation was more significant for the Treg subset (Figure 5G), which showed higher pSTAT5 levels following stimulation compared to negative control also in all patients with CLL (Figure 5C).

Therefore, antigen-stimulated pSTAT5 levels in Tregs better reflected results obtained using the traditional method of antigen-specific T cell analysis (AIM) than pSTAT5 response in Tcons. However, using our novel Treg-specific pSTAT5 assay, the response to stimulation was found in the FOXP3+ subset of CD4^+^ T-cells also from patients with advanced CLL, whose AIM response was absent.

### 3.4. Basal STAT5 Phosphorylation in the FOXP3+ Subset Is Significantly Increased in CLL Patients Treated with Chemoimmunotherapy

STAT5 signaling is implicated in the homeostasis of FOXP3+ Treg cells [39], which was shown before to be expanded in patients with advanced CLL [14,15].

As we found responses to stimulation in the FOXP3+ subset of CD4^+^ T-cells also from patients with advanced CLL, we compared basal/unstimulated levels of STAT5 phosphorylation between the CD4^+^ T-cell subsets in whole blood samples from a larger group of patients with CLL and healthy controls (HC). Patients with advanced disease before therapy and patients treated with ibrutinib or CIT were included. When compared to both HC and untreated patients, the differences in pSTAT5 levels (Δ pSTAT5 MFI) between FOXP3+ cells and the Tcon subset of CD4^+^ T-cells were higher, but not significantly different, in treated patients with CLL in general. However, the FOXP3+ subset displayed significantly higher pSTAT5 levels relative to Tcon cells in patients treated with CIT than in patients treated with ibrutinib, untreated CLL patients with advanced disease, or HCs (Figure 5H).

As ibrutinib treatment modulates not only B-cell, but also T-cell activation [40], STAT5 phosphorylation in CD4^+^ T-cells could also be affected/suppressed in patients treated with this BTK inhibitor. This did not seem to be the case in the FOXP3+ subset from some patients treated with ibrutinib, as their pSTAT5 levels were much higher than in HCs and untreated patients with advanced CLL (Figure 5H,I).

Collectively, the results of both in vitro as well as antigen-specific pSTAT5 assays have shown higher responses to stimulation in the FOXP3+ subset, which was characterized by significantly higher levels of basal pSTAT5 relative to Tcon cells in patients with advanced CLL treated with CIT.

## 4. Discussion

Basic, as well as clinical, research [41,42,43] has focused on the study of Treg cells for many years due to their unique function, which is the suppression of immune responses. Most methods to analyze the suppressive capacity of Treg cells in vitro aim to measure inhibition of effector Tcon proliferation, although cytokine production may also be used as the read-out [23,44]. Here, we present a protocol for the combined detection of proliferation, via BrdU-FITC incorporation, and monitoring of cytokine signaling via phosphorylated STAT5 within FOXP3+ and FOXP3− Tcon subpopulations in response to polyclonal anti-CD3/CD28 stimulation. Furthermore, a second method is presented here that allows for the detection of cytoplasmic vs. nuclear localization of transcription factors and phosphorylated STAT proteins in FOXP3+ and FOXP3− subsets of lymphocytes following cytokine stimulation ex vivo in whole blood samples. Finally, a whole blood protocol for the detection of STAT5 phosphorylation in response to SARS-CoV-2 antigen-specific stimulation is presented, which also allows for comparison of signaling responses in FOXP3+ Treg and Tcon subpopulations.

All described, flow cytometry-based methods provide a clear advantage over other techniques, such as Western blotting, or other antibody-based technologies, such as immunohistochemistry, enzyme-linked immunosorbent assay (ELISA), protein array, and reverse phase protein array (RPPA). First, by exploiting the multiparameter capacity of flow cytometry, signaling pathways can be analyzed simultaneously with proliferation in different subsets of a heterogeneous cell population, such as peripheral blood T-cells, during the in vitro assay. Furthermore, these methods combined with multispectral imaging cytometry allow for more in-depth analysis of cell signaling at a single-cell level, including translocation of transcription factors from the cytoplasm to the nucleus in specific, and even rare, subpopulations such as FOXP3+ lymphocytes. In addition, cells in the whole blood antigen-specific pSTAT5 assay do not have to be purified prior to analysis, allowing not only for a high-throughput analysis, but also for identification of subtle STAT5 phosphorylation responses in Tregs from CLL patients treated with immunosuppressive drugs. Finally, as we also show on example of FOXP3+ cells from patients with CLL, using such novel phosflow methods, increased basal STAT signaling, possibly driving perturbed homeostasis of Tregs, can be identified.

Using these phosphor-specific flow cytometry and multispectral imaging cytometry methods, several additional observations were made.

The homeostatic cytokine IL-2, which acts on Tregs to maintain them in a functional state [45,46], was probably mostly derived from conventional CD4^+^ cells responding to CD3/CD28 stimulation in our in-vitro assay, as FOXP3+ cells were characterized by significantly lower intracellular IL-2 production. In addition, intracellular IL-2 expression and STAT5 activation were also almost completely mutually exclusive. Recent results from a mouse model suggest that IL-2 production by Tcon cells comes at a cost of loss of sensitivity to homeostatic STAT5 signaling [47]. In the same study, IL-2 production was reliably detected in FOXP3+ Tregs in vivo. However, the titrated response of Tregs to the autocrine production of IL-2 suggested that Tregs are more responsive to overall levels of IL-2 production than to autocrine sources [47]. In addition, Treg cells are also known to respond differently than Tcon cells to IL-2 as they express IL-2Rα, even when lacking antigenic stimulation [31]. Consistent with that, FOXP3+ cells displayed significantly higher levels of STAT5 activation when compared to Tcon cells in a coculture of CD4^+^CD25^−^ with CD4^+^CD25^+^ cells, pointing to their higher responses to the same level of the homeostatic cytokine in our in vitro assay.

The protocol of our in vitro assay includes staining with a dye that binds to total DNA (7-AAD), which is coupled with immunofluorescent BrdU staining. With this combination, two-color flow cytometric analysis permits the enumeration and characterization of cells that are actively synthesizing DNA (BrdU incorporation) in terms of their cell cycle position (ie, G0/1, S, or G2/M phase defined by 7-AAD staining intensities) [48,49]. Supplementary Figure S7 shows gating and the representative pSTAT5 levels at the mentioned cycle phases. However, methods based on DNA binding dyes, such as propidium iodide and 7-AAD, are insufficient to understand the detailed cell cycle status because DNA content alone cannot distinguish resting/quiescent cells (G0) from G1 phase cells. Some alternative methods have been developed to overcome this limitation, including staining for nuclear proliferation antigens, such as Ki-67 [50,51].

In line with the findings of Bitar et al., which clearly showed that upon CD3/CD28 activation, T-cell proliferation strongly depends on the phosphorylation of STAT5 [52], suppression of Tcon cell cycle progression was accompanied by a significant decrease of pSTAT5 in our in vitro assay. We found a dependency of STAT5 phosphorylation on IL-2 levels after CD3/CD28 stimulation in FOXP3+ as well as FOXP3− Tcon cells, as pSTAT5 was significantly decreased after incubation with neutralizing IL-2 antibodies in both subsets. However, the decrease in pSTAT5 was more significant in FOXP3+ cells, again pointing to their higher sensitivity to IL-2.

A recent study, which did not include analysis of FOXP3 expression, has shown that only in a population of “conditioned” competent T-cells bearing the CD25 in response to IL-2, JAK3/STAT5 signaling via the high-affinity IL-2Rαβγ_c_ is augmented to maintain the sustained IL-2Rα expression as well as cell proliferation [53]. By simultaneous analysis of pSTAT5 and FOXP3 expression, we could show that CD25^+^ Tcon cells displayed higher pSTAT5 levels than CD25^−^ Tcon cells. However, FOXP3+ cells have shown not only higher CD25 expression, but also significantly higher pSTAT5 levels even when compared to CD25^+^ Tcon cells after anti-CD3/CD28 stimulation. Therefore, higher levels of pSTAT5 in FOXP3+ cells than in Tcon cells, found also in coculture of CD4^+^CD25^−^ with CD4^+^CD25^+^ cells, suggests that FOXP3+ cells in conditions of our in vitro assay retain their higher sensitivity to IL-2, which is one of the most important characteristics of Treg cells [31]. However, to analyze Treg suppression, our in vitro assay would have to be modified to include quantification of Treg cells, which is not based solely on FOXP3 expression. As shown in the study-flow diagram (Figure 1), the purity of magnetically-selected CD4^+^ T-cells and their CD25^+^/CD25- subsets was analyzed only in preliminary experiments, but not for every sample before our in vitro assay.

Although in the original description of the in vitro suppression assay, CD4^+^CD25^+^ cells were isolated with the magnetic microbead procedure [23], the issue of how best to purify human Treg cells is complicated not only by the difficulty of isolating CD25^hi^ Treg cells using a magnetic bead-based methodology [54], but also by heterogeneity within the CD25^hi^ population [55]. Therefore, despite the fact that both significantly higher levels of CD25 expression after CD3/CD28 stimulation as well as significantly higher levels of IL-2-dependent pSTAT5 were shown for FOXP3+ cells in our in vitro assay, a combination of the described methods with flow cytometry sorting could offset some of the shortcomings of the magnetic microbead procedure. However, a recent evaluation of T-cell functionality revealed activation of p38 MAPK signaling pathways in T-cells also after flow cytometry sorting [56]. Therefore, in cases where negative selection is not possible, isolated cells are distinct to their previous characteristics, as shown also for Treg cells and their in vivo functionality, which is enhanced by the release of isolation reagents [57,58].

Thus, our aim was to analyze Treg signaling without prior cell separation in a microenvironment as close as possible to the physiological/in vivo condition. We therefore analyzed whole blood samples ex vivo, either to evaluate basal STAT5 signaling, or responses to stimulation with SARS-CoV-2-specific antigens and with recombinant human IL-2. Our results indicate that STAT5 nuclear translocation events within FOXP3+ and FOXP3− cells can be compared and quantified by correlating transcription factor and nuclear images collected in flow using the imaging flow cytometer. Differential dose-response effects on CD4^+^FOXP3− Tcon cells versus Treg of recombinant human IL-7 and IL-2 in the ex vivo whole blood pSTAT5 assay were shown before [59]. However, combining ex vivo whole blood IL-2 stimulation with imaging cytometry in our assay also confirmed higher levels of nuclear localization of pSTAT5 in FOXP3+ than in FOXP3− cells.

To analyze Treg STAT5 signaling in response to antigen-specific stimulation ex vivo, we have chosen commercially available tubes from the whole blood Interferon-γ Release immune Assay (IGRA), which uses mixtures of SARS-CoV-2 S proteins selected to activate both CD4^+^ and CD8^+^ T-cells. A good correlation between T cellular responses detected by the QuantiFERON SARS-CoV-2 assay with T-cells expressing activation-induced markers (AIMs) has been demonstrated in healthy individuals 8 weeks after the second dose of the BNT162b2 mRNA vaccine [60]. In addition, the frequency of AIM-T cells was also detected in the blood coming from Quantiferon SARS-CoV-2 kit test tubes to define SARS-CoV-2-specific T cells after a booster dose of the BNT162b2 mRNA-based vaccine [61].

We found a significant correlation between the results of the OX40/CD25 AIM assay, which was previously shown to be highly efficient at detecting antigen-specific CD4 T-cells [62], and pSTAT5 levels in both Treg and Tcon subsets in antigen-stimulated, but not in control samples. However, the correlation was more significant for the Treg subset, which displayed significantly higher responses to stimulation than Tcon cells, and also in the antigen-specific pSTAT5 assay. These results indicate that Treg-specific pSTAT5 responses detected in our whole blood antigen-specific assay could be used for quantification of spike-specific T-cell responses, producing results that are comparable to those obtained with the well-established AIM assay used to analyze T-cell responses. As both in vitro as well as ex-vivo assays have shown higher levels of IL-2-dependent signaling in Tregs, their pSTAT5 levels could serve as a more sensitive read out of exposure to IL-2, produced during antigen-specific activation of Tcons. Consistent with that, antigen-stimulated pSTAT5 levels in Tregs, but not in Tcons, were increased above unstimulated in samples from all healthy donors, as well from CLL patients with advanced disease.

T-cells are especially critical in immune protection against SARS-CoV-2 in CLL patients who undergo therapy with B-cell depleting agents, such as anti-CD20 antibody, as part of their CIT. It was reported that CLL patients with diminished numbers of functional CD19+ B-cells, a key player in the humoral response against the SARS-CoV-2 virus, developed T-cell immune responses to COVID-19 vaccination, which were, however, dependent on the current treatment status [12]. In addition, in a recent study, which used the Quantiferon IGRA assay to measure responses to SARS-CoV-2 antigen-specific stimulation following vaccination, the cellular response was absent in patients with high amounts of B-cells in ibrutinib-treated CLL groups, suggesting that the generation of the cellular immune response to the vaccine is hampered by the disease burden [13]. Consistent with that, in both our patients with CLL characterized by high disease burden/advanced Binet stage, responses were much lower compared to healthy donors in our novel antigen-specific Treg pSTAT5 assay. In addition, when tested with the AIM assay, the percentage of OX40+CD25^+^ cells did not increase above unstimulated in samples from some CLL patients with advanced disease on CIT. However, the antigen-stimulated pSTAT5 response was still detected in FOXP3+ Tregs, but not Tcon cells, from the same patients. Therefore, basal/unstimulated pSTAT5 levels were in a larger group of patients with advanced CLL measured ex vivo in the FOXP3+ and FOXP3− subset of CD4 T-cells to evaluate the response of each subset to its endogenous cytokine milieu. Although differences in basal pSTAT5 levels between the two subsets were much higher in some patients treated with ibrutinib compared to both healthy controls and untreated patients with advanced CLL, a significant increase in ∆pSTAT5 relative to Tcon cells was found only in the FOXP3+ subset of CD4^+^ T-cells from patients treated with CIT.

Collectively, our results show that the described novel methods allow for the identification of unique STAT signaling characteristics of Treg cells in vitro as well as ex vivo in response to both cytokine and antigen-specific stimulation. Treg pSTAT5, induced by SARS-CoV-2 antigen-specific stimulation, which was found even in patients with advanced CLL, could be used for the sensitive detection of spike-specific T-cell responses. On the other hand, due to the critical role of STAT5 signaling in Treg development, significantly higher levels of basal pSTAT5 in the FOXP3+ subset relative to FOXP3− Tcon cells, found in patients treated with CIT, could be responsible for the perturbed homeostasis between the Treg and Tcon subset of CD4^+^ T-cells. Thus, we speculate that, through the use of this novel tool, the efficacy of immunosuppressive drugs and signaling inhibitors and their possible off target effects can be assessed, enabling optimization of the therapy for individual patients.

## Figures and Tables

**Table 1:** Characteristics of patients with CLL in the study.

	Gender	Age	Disease Stage	Therapy
Patient 1	male	35	Binet C	CIT *
Patient 2	male	62	Binet C	BTKi *
Patient 3	female	69	Binet C	BTKi *
Patient 4	male	79	Binet C	CIT *
Patient 5	male	84	Binet C	BTKi *
Patient 6	female	69	Binet C	CIT *
Patient 7	female	83	Binet C	CIT *
Patient 8	female	61	Binet B	BTKi *
Patient 9	male	77	Binet C	CIT *
Patient 10	male	76	Binet C	CIT *
Patient 11	female	63	Binet C	BTKi *
Patient 12	male	63	Binet C	CIT
Patient 13	female	63	Binet C	CIT
Patient 14	male	89	Binet C	BTKi
Patient 15	male	59	Binet C	CIT
Patient 16	female	66	Binet C	CIT
Patient 17	male	74	Binet C	CIT
Patient 18	male	71	Binet C	BTKi
Patient 19	male	63	Binet C	BTKi
Patient 20	male	69	Binet C	BTKi
Patient 21	male	77	Binet A	0
Patient 22	female	82	Binet C	CIT
Patient 23	male	83	Binet C	CIT
Patient 24	male	61	Binet B	0
Patient 25	female	78	Binet C	BTKi
Patient 26	male	71	Binet A	0
Patient 27	male	83	Binet A	0
Patient 28	male	56	Binet C	CIT *
Patient 29	male	81	Binet C	BTKi
Patient 30	male	66	Binet C	CIT *

Abbreviations: CIT, chemo-immunotherapy; BTKi, Bruton Tyrosine Kinase inhibitor; * samples analyzed also before therapy.

## Data Availability

Datasets used in this article are available from corresponding author on reasonable request.

## Supplementary Materials

The following supporting information can be downloaded at: https://www.mdpi.com/article/10.3390/bios13050539/s1, Figure S1: Ancestry gating for Figure 2A; Figure S2: Intracellular IL-2 expression in the FOXP3+ and FOXP3− subset of CD4^+^ T-cells.; Figure S3: pSTAT5 levels in the IL-2+ vs. IL-2- subset of FOXP3− Tcon cells.; Figure S4: Representative dot plot of pSTAT5 vs. intracellular IFNg expression.; Figure S5A,B: Staining with FMO (Fluorescence Minus One) controls and pSTAT5 signaling responses (pSTAT5 MFI fold change) of FOXP3+ and FOXP3- Tcon subsets of CD4^+^ T-cells from healthy controls and patients with CLL to Ag-specific stimulation. Representative histograms of pSTAT5 in both the FOXP3− and FOXP3+ subsets of CD4^+^ T-cells from the healthy donor for stimulated and unstimulated cells are shown in panel B.; Figure S6A,B: Levels of fluorescence signals (MFI) of FMO negative controls in the FOXP3+ and FOXP3− subset for antigen stimulated cells from patients with CLL and representative histograms of FMO negative controls and pSTAT5 in both FOXP3− and FOXP3+ subsets of CD4^+^ T-cells from the patient with CLL for stimulated and unstimulated cells.; Figure S7: Gating and the representative pSTAT5 levels in G0/G1, S, and G2/M phases of cell cycle.
